# Social Participation and the Prevention of Decline in Effectance among Community-Dwelling Elderly: A Population-Based Cohort Study

**DOI:** 10.1371/journal.pone.0139065

**Published:** 2015-09-25

**Authors:** Kimiko Tomioka, Norio Kurumatani, Hiroshi Hosoi

**Affiliations:** Nara Prefectural Health Research Center, Nara Medical University, Kashihara, Nara, Japan; Banner Alzheimer's Institute, UNITED STATES

## Abstract

**Background:**

We examined the association between a decline in effectance and social participation (SP) from the perspective of the number and the type of SP in a prospective cohort study.

**Methods:**

Included in this analysis were community-dwelling elderly aged ≥65 without dependency on the basic activities of daily living and reporting a perfect baseline effectance score (n = 4,588; mean age 72.8±5.7). SP was categorized into 5 types: neighborhood associations, hobby groups, local event groups, senior citizen clubs, and volunteer groups. Effectance was evaluated using the Tokyo Metropolitan Institute of Gerontology Index of Competence. Using logistic regression analysis, odds ratio (OR) and a 95% confidence interval (CI) for a decline in effectance were calculated. Age, family, BMI, pensions, medical history, medications, alcohol, smoking, cognitive function, depression, social support, ADL, and IADL were used as covariates.

**Results:**

During the 3-year follow-up, 17.8% of eligible participants reported a decline in effectance. After adjustment for covariates, participation in various groups was associated with the preservation of effectance for both genders. Regarding the type of SP, among females, participation in neighborhood associations (OR: 0.62, 95%CI: 0.48–0.81), hobby groups (0.58, 0.43–0.77), local event groups (0.63, 0.47–0.86), and volunteer groups (0.53, 0.35–0.82) was inversely associated with a decline in effectance. Among males, the beneficial effect was more likely limited to hobby groups (0.59, 0.43–0.81) and volunteer groups (0.57, 0.39–0.83).

**Conclusions:**

Our results suggest that participation in a variety of social groups is effective for maintenance of older people’s effectance, while the beneficial effect of each type of SP on effectance is stronger for females than for males. Recommending community-dwelling elderly to participate in social groups appropriate for their gender may be effective for successful aging.

## Introduction

The World Health Organization has proposed autonomy in functional capacity as a proxy of health for the elderly [1]. There are various levels of functional capacity. Lawton developed a hierarchical model of competence comprising seven sublevels: life maintenance, functional health, perception and cognition, physical self-maintenance (corresponding to activities of daily living; ADL), instrumental self-maintenance (corresponding to instrumental activities of daily living; IADL), effectance, and social role [2]. Based on Lawton’s model, Koyano and colleagues developed the Tokyo Metropolitan Institute of Gerontology Index of Competence (TMIG-IC) [3]. The TMIG-IC was designed to measure three higher-level functional capacities. Higher-level competence, which indicates a higher-level functional capacity above basic ADL, corresponds to the 5th, 6th, and 7th sublevels of Lawton’s hierarchical model. Among the three sublevels of higher-level functional capacity, effectance represents the motivation behind human needs to create tension, explore, and vary the environmental psychological field, such as having an interest in health-related information through the mass media [2]. Earlier prospective cohort studies have reported that poor effectance is a risk factor for cognitive impairment [4,5], and that good effectance is associated with remaining independence in IADL for the nondisabled older people living in a community [5,6]. Since maintaining the ability of effectance can prevent future deterioration in cognitive function and IADL, it is imperative to identify the modifiable protective factors for effectance in elderly adults.

Social participation, which is a source of social relations and describes a person’s participation in formal and informal group activities [7,8], has long been recognized as an essential component of active aging [9]. Social participation declines as a result of the normal aging process [10,11] and may correlate with better daily function and less disability in old age. Some prior longitudinal studies of social participation in elderly persons have focused on an outcome referred to as functional disability, but the results of these studies have been contradictory, with studies reporting positive associations [12–16], associations only in certain age ranges [16,17], or only one gender [18–21], and even negative associations [22]. In addition, many studies on social participation have focused on ADL [13–15,17–20,22], and limited data are available for IADL [12,16,21]. To the best of our knowledge, no studies have been conducted on the relationship between effectance and social participation among community-dwelling elderly.

Studies that examined the effects of social participation according to different types of groups showed that participation in several groups may have a protective effect on depression [23,24], well-being [25], and functional disability [13]. In contrast, other studies examining the effects of participation in civic groups on all-cause mortality [26,27], cognitive function [27], and oral health status [28] have not always shown a reduction in risk with participation. These above-mentioned studies [13,23–28] suggest that the relationship of social participation with effectance may vary depending on the number and type of social participation, a possibility that has yet to be studied. Since social participation can be enhanced [29], clarifying what forms of participation (i.e., participation in all or particular types of social groups) are effective in the maintenance of effectance can provide a better understanding for increasing the healthy life years of the elderly.

In the present study, we set out to examine the relationship between a decline in effectance and social participation from the perspective of the number of types of groups participated in and the type of social participation in a prospective cohort study. Our study hypothesis is that community-dwelling elderly who participate in a variety of social groups are less likely to experience a decline in effectance compared with those who do not participate in social groups. Because functional capacity can influence social participation [30], to examine the impact of social participation on effectance, our analyses were restricted to persons reporting intact effectance at baseline. Additionally, as prior studies have identified possible gender differences in the effect of social participation on health outcomes [18–21,24, 31,32], we were interested in studying the possible gender differences in the association between social participation and a decline in effectance.

## Methods

### Study area and subjects

**Fig 1:**
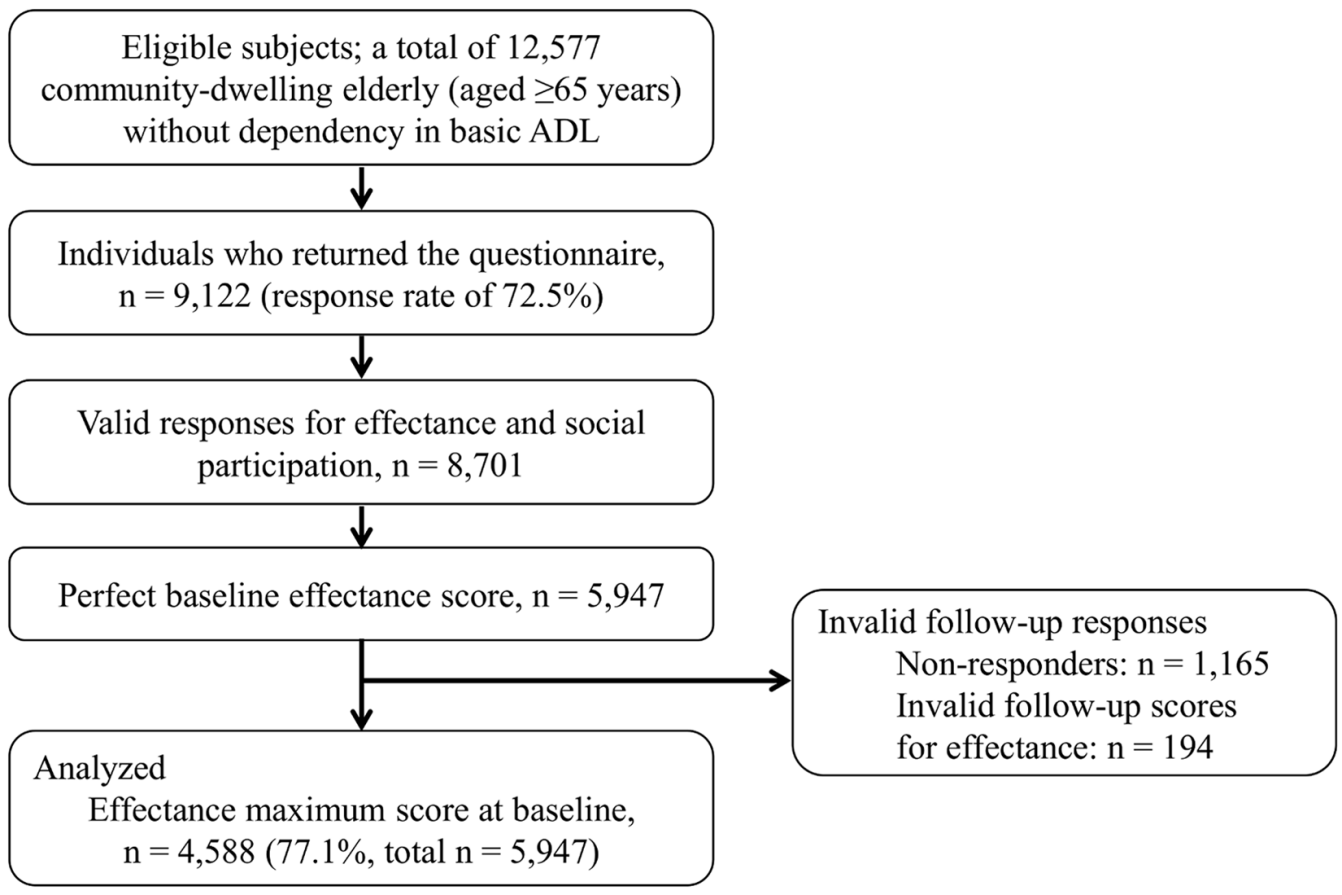
Flow diagram of the enrollment of study participants. ADL = activities of daily living.

The target area for this study was Kashiba City, located in the mid-west part of Nara prefecture. At the end of January, 2011, the total population was 76,173, the total area 24.23 km^2^, and the rate of the aging population 17.5%. This city is a typical commuter town of the megacity, Osaka. Eligible subjects were community-dwelling individuals aged 65 years and older who were not certified as ‘dependent in basic activities of daily living’ by the public long-term care insurance system (e.g., care levels 3–5). In January 2011, the city office mailed the baseline questionnaires to 12,577 community-dwelling older adults (response rate of 72.5%). Fig 1 displays the flow diagram of the enrollment of study participants. Among the 9,122 (4,198 males and 4,924 females) persons who participated in the baseline survey, we excluded 421 individuals because of an invalid response for effectance and/or social participation. Thus, 8,701 valid responses were obtained. Compared to persons with valid responses, subjects without valid responses showed significantly older and poorer ADL, but no difference by gender (Table 1). Of the baseline population, 5,947 had a maximum score for the effectance indices. Similar postal questionnaire surveys were conducted 3 years later to obtain follow-up data. A total of 4,782 (80.4%, of 5,947) people returned the second questionnaires. After excluding individuals with invalid follow-up scores (n = 194), 4,588 participants were analyzed. Persons with invalid follow-up responses were significantly older, more male, and had poorer ADL than those with valid follow-up scores (Table 1). All study participants provided signed informed consent. This study protocol was approved by the Nara Medical University Ethics Committee (approval number 939).

**Table 1:** Basic attributes of subjects with or without valid response for baseline and follow-up data.

Basic attributes at baseline	Baseline data				P-value^b^	Follow-up data				P-value^b^
	Valid response		Invalid response^a^			Valid response		Invalid response^c^		
	(n = 8,701)		(n = 421)			(n = 4,588)		(n = 1,359)		
75 years of age and older	3630	(41.7)	258	(61.3)	< 0.001	1542	(33.6)	598	(44.0)	< 0.001
Male	4010	(46.1)	188	(44.7)	0.582	2124	(46.3)	693	(51.0)	0.002
Subjects with partial dependency in their activities of daily living^d^	839	(9.6)	69	(16.4)	< 0.001	119	(2.6)	80	(5.9)	< 0.001

Data are given as n (%).

^a^ Subjects with invalid response for effectance and social participation.

^b^ Differences between subjects with or without valid response were analyzed using Fisher's exact test.

^c^ Subjects who did not participate the follow-up survey or were invalid response for effectance at follow-up.

^d^ Subjects who were certified as support levels 1–2 or care levels 1–2 by public long-term care insurance system.

### Assessment of effectance

Effectance was measured using the subscale of the TMIG-IC [3]. The TMIG-IC is a widely used standard in Japan, and its reliability and validity have been tested [33]. This is a multidimensional 13-item index (Table 2). Items 1–5 are classified as IADL, items 6–9 are classified as effectance, and items 10–13 are classified as social role. The response to each item was designed simply as ‘yes’ (able to do) or ‘no’ (unable), and scored 1 for each ‘yes’ and 0 for each ‘no’. Scores for the effectance subscale range from 4 (i.e., highest level) to 0 (i.e., lowest). For the effectance measure, a change from a maximum score at baseline to anything less than a maximum score at the 3-year follow-up was defined as decline [5,6]. Thus, participants with maximum baseline scores on effectance were divided into two groups according to effectance score at follow-up: decline (<4) and no decline (4 points).

**Table 2:** The Tokyo Metropolitan Institute of Gerontology Index of Competence (TMIG-IC) for assessing higher-level functional capacity in older adults.

Sub-scales	Questionnaires	
Instrumental Activities of Daily Living (IADL)		
1 Can you use public transportation (bus or train) by yourself?	1. Yes	0. No
2 Are you able to shop for daily necessities?	1. Yes	0. No
3 Are you able to prepare meals by yourself?	1. Yes	0. No
4 Are you able to pay bills?	1. Yes	0. No
5 Can you handle your own banking?	1. Yes	0. No
Effectance		
6 Are you able to fill out forms for your pension?	1. Yes	0. No
7 Do you read newspapers?	1. Yes	0. No
8 Do you read books or magazines?	1. Yes	0. No
9 Are you interested in news stories or programs dealing with health?	1. Yes	0. No
Social Role		
10 Do you visit the homes of friends?	1. Yes	0. No
11 Are you sometimes called on for advice?	1. Yes	0. No
12 Are you able to visit sick friends?	1. Yes	0. No
13 Do you sometimes initiate conversations with young people?	1. Yes	0. No

### Assessment of social participation

Social participation was defined as the person’s involvement in social groups. For the purposes of this study, social participation was classified into five types: neighborhood associations, hobby groups, local event groups, senior citizen clubs, and volunteer groups. Subjects were given a choice between ‘currently participate’ or ‘do not participate’ for each type of social group. The total number of types of social groups in which each subject participated was tallied and participation was categorized as 0 (no participation), 1, 2 or ≥3 groups.

### Covariates

Based on previous studies [4–6,34], age, gender, family structure, body mass index (BMI), pensions, self-reported medical conditions, and the number of medications used were used as covariates that may correlate with social participation and incident effectance decline. Since behavioral, psychosocial, and physiological pathways may be potential mechanisms for social participation to influence health [35], these factors were also used as covariates. Alcohol consumption and smoking were used as behavioral factors. Cognitive function, depression, and social support were used as psychosocial factors. ADL and IADL were used as physiological factors.

Age was used as continuous variables. Family structure was categorized as living alone, living with only spouse, living with a person other than spouse, and living with three or more persons. BMI was categorized as underweight (i.e., <18.5), normal (i.e., 18.5-<25.0), and overweight (i.e., ≥25.0). Pensions were used as an indicator of socioeconomic status. Pensions were categorized as national pension, employees' pension, mutual aid association pension, and other (e.g., disability or survivor pension). Current medical history was measured by the question, “Do you receive treatment now?” to which respondents answered “yes” or “no”. The number of comorbidities (hypertension, stroke, heart diseases, diabetes mellitus, chronic respiratory disease, musculoskeletal disorder, otological disease, ophthalmologic disease, and cancer) present at baseline was categorized as zero, one, and ≥2. The number of medications used was categorized as none, 1–2, 3–4, or more than 5. Alcohol consumption was categorized as nondrinkers, social drinkers, occasional drinkers, and daily drinkers. Smoking was categorized as never-smokers, ex-smokers, and current smokers. Cognitive function was evaluated using the Cognitive Performance Scale (CPS: score range 0–6) [36], and a score ≥1 was considered indicative of the presence of poor cognitive functioning. Depression was evaluated using the 5-item short form of Geriatric Depression Scale (GDS-5: score range 0–5) [37], and a score ≥2 was defined as the presence of depression. Social support was measured by receiving emotional and instrumental support (available vs. not available) [13]. ADL was evaluated using the Barthel index (score range 0–100) [38], and a score <100 was defined as subjects with poor ADL. IADL was categorized as 5 full mark (independent) and <5 points (dependent), based on their TMIG-IC subscale scores [39].

### Statistical Analysis

Data comparisons between those with valid responses and those without valid responses or those with a decline in effectance and those without a decline were tested with Fisher’s exact test. Logistic regression analysis was carried out using ‘with or without a decline of effectance’ (effectance score <4 or full mark at follow-up) as a dependent variable. The independent variable was social participation at baseline (‘the number of social groups’ and ‘the type of social participation’). The results were shown as odds ratio (OR) with 95% confidence interval (CI). In each model, non-participation in a social group was set as the referent category. In the univariate model (Model 1), we calculated the crude OR for a decline in effectance based on the number of social groups and the type of social participation. In the multivariable model (Model 2), regression analysis was performed with simultaneous forced entry of age, family structure, BMI, pensions, the number of comorbidities, the number of medications used, alcohol, smoking, cognitive function, depressive symptoms, social support, ADL, and IADL as covariates. Goodness of fit was assessed on the basis of the technique of Hosmer and Lemeshow [40]. For the association of the number of social groups with a decline in effectance, a linear trend test was also conducted to assess its dose–response relationship. To examine whether the relation between social participation and a decline in effectance varied by gender, we performed logistic regression analysis stratified by gender. The level of significance was 0.05 (two tailed). Statistical analyses were performed using SPSS (version 17.0; SPSS Japan Inc., Tokyo, Japan).

## Results

The mean age of the study population at baseline was 72.8 ± 5.7 (range 65–95), 46.3% were male, and 17.8% declined in effectance over the 3-year follow-up period. Of all respondents, 35.2% did not participate in any group, 30.7% participated in one group, 17.7% in 2 groups, and 16.4% in ≥3 groups. Of the respondents, 34.1% participated in neighborhood associations, 25.0% in hobby groups, 25.0% in local event groups, 22.1% in senior citizen clubs, and 13.8% in volunteer groups.

Table 3 shows the baseline characteristics according to subjects with and without decline in effectance. Compared to those without effectance decline, those with effectance decline were more likely to be older, receive national pension, have more comorbidities, use more medications, have poorer cognitive function, depression, lower social support, and dependency in ADL and IADL, and less likely to participate in social groups. Alternatively, gender, family structure, BMI, behavioral factors, and participation in senior citizen clubs were not significantly different between the two groups.

**Table 3:** Characteristics of study participants at baseline stratified according to the decline in effectance during the 3-year follow-up

Baseline Characteristic	Non-decline group		Decline group		P-value^a^
	(n = 3,771)		(n = 817)		
Socio-demographic					
Age: 75 years and older	1,164	(30.9)	378	(46.3)	<0.001
Gender: Male	1,736	(46.0)	388	(47.5)	0.462
Family structure: living alone	348	(9.2)	84	(10.3)	0.355
Body mass index: normal	2,804	(74.4)	610	(74.7)	0.825
Pension: national pension	1,304	(34.6)	314	(38.4)	0.039
The number of chronic medical conditions					
Two or greater	1,051	(27.9)	279	(34.1)	0.001
The number of medications used					
More than 5	648	(17.2)	203	(24.8)	<0.001
Behavioral factors					
Alcohol intake: daily drinker	968	(25.7)	192	(23.5)	0.214
Smoking history: current smoker	300	(8.0)	72	(8.8)	0.437
Psychosocial factors					
Poor cognitive function: CPS ≥1	376	(10.0)	173	(21.2)	<0.001
Depression: GDS ≥2	566	(15.0)	221	(27.1)	<0.001
No social support	264	(7.0)	108	(13.2)	<0.001
Physiological factors					
Poor ADL: Barthel-index <100	647	(17.2)	208	(25.5)	<0.001
Poor IADL: TMIG-IC <5	210	(5.6)	98	(12.0)	<0.001
Social participation					
The number of social groups					
Zero	1,234	(32.7)	380	(46.5)	<0.001
One	1,183	(31.4)	226	(27.7)	
Two	689	(18.3)	125	(15.3)	
Three or greater	665	(17.6)	86	(10.5)	
Type of social participation (subjects with participation of each group)					
Neighborhood associations	1,351	(35.8)	214	(26.2)	<0.001
Hobby groups	1,025	(27.2)	122	(14.9)	<0.001
Local event groups	999	(26.5)	148	(18.1)	<0.001
Senior citizen clubs	821	(21.8)	194	(23.7)	0.227
Volunteer groups	572	(15.2)	62	(7.6)	<0.001

Data are given as n (%).

ADL, activities of daily living; CPS, Cognitive Performance Scale; GDS, Geriatric Depression Scale; IADL, instrumental activities of daily living; TMIG-IC, Tokyo Metropolitan Institute of Gerontology Index of Competence.

^a^ p values were calculated using Fisher's exact test.

Table 4 shows the results of the univariate and multivariate logistic regression analyses of social participation and effectance decline by gender. For males, after no adjustment (Model 1), there was a significant dose-response relationship between more participation in social groups and lower effectance decline (p for trend < .001), and participating in ≥1 groups was associated with significantly lower odds of effectance decline compared with non-participation. After adjustment for covariates (Model 2), the dose-response relationship remained significant (p for trend = .009), but the OR for effectance decline among those participating in 2 groups was not statically significant (OR = 0.80, 95% CI = 0.58–1.12). Regarding the type of social participation, after no adjustment (Model 1), participation in all but senior citizen clubs was associated with a decreased risk of effectance decline. After adjustment for covariates (Model 2), the social groups significantly associated with the prevention of effectance decline were limited to hobby groups (OR = 0.59, 95% CI = 0.43–0.81) and volunteer groups (0.57–0.39–0.83). For females, in the crude model (Model 1), a significant dose-response relationship was seen, with progressively lower ORs as the number of different types of social groups increased (p for trend < .001), and participating in ≥1 groups was significantly associated with reduced risk of developing decline in effectance. In Model 2, where the data was adjusted for covariates, these associations were attenuated but remained basically unchanged: the ORs for effectance decline in subjects with participation in one group, two groups, and ≥3 groups were 0.69 (95% CI = 0.53–0.89), 0.62 (0.45–0.87), and 0.41 (0.28–0.61), respectively, compared to subjects with nonparticipation in any social group (p for trend < .001). Regarding the type of social participation, participation in all social groups except for senior citizen clubs was inversely associated with effectance decline without any covariate adjustment (Model 1). After adjustment for covariates (Model 2), the ORs for the types of social participation that were observed in Model 1 tended towards 1.00, but significant associations were not changed: neighborhood associations (OR = 0.62, 95% CI = 0.48–0.81), hobby groups (0.58, 0.43–0.77), local event groups (0.63, 0.47–0.86), and volunteer groups (0.53, 0.35–0.82). Hosmer-Lemeshow analysis provided no evidence of lack of fit (p ≥0.05 in all cases).

**Table 4:** Odds ratios (95% confidence interval) for 3-year decline in effectance by gender.

	Males (n = 2,124)					Females (n = 2,464)				
	Model 1		Model 2			Model 1		Model 2		
	Crude OR & 95% CI		Adjusted OR & 95% CI		Goodness of fit^a^	Crude OR & 95% CI		Adjusted OR & 95% CI		Goodness of fit^a^
**The number of types of social groups**										
Zero	1.00		1.00		p = 0.58	1.00		1.00		p = 0.64
One	0.62	0.47–0.82	0.68	0.51–0.91		0.62	0.48–0.79	0.69	0.53–0.89	
Two	0.70	0.52–0.96	0.80	0.58–1.12		0.50	0.36–0.68	0.62	0.45–0.87	
Three or greater	0.53	0.37–0.74	0.60	0.42–0.87		0.33	0.23–0.48	0.41	0.28–0.61	
Test for linear trend	p <0.001		p = 0.009			p <0.001		p <0.001		
**Type of social participation (reference: non-participation of each social group)**										
Neighborhood associations	0.78	0.62–0.98	0.90	0.71–1.15	p = 0.73	0.49	0.38–0.64	0.62	0.48–0.81	p = 0.78
Hobby groups	0.51	0.37–0.69	0.59	0.43–0.81	p = 0.64	0.45	0.34–0.59	0.58	0.43–0.77	p = 0.36
Local event groups	0.74	0.57–0.96	0.82	0.63–1.08	p = 0.85	0.49	0.37–0.66	0.63	0.47–0.86	p = 0.54
Senior citizen clubs	1.06	0.80–1.41	0.91	0.67–1.24	p = 0.59	1.17	0.93–1.48	0.88	0.68–1.13	p = 0.18
Volunteer groups	0.50	0.35–0.72	0.57	0.39–0.83	p = 0.66	0.41	0.27–0.62	0.53	0.35–0.82	p = 0.58

OR, odds ratio; CI, confidence interval.

^a^ Goodness of fit determined by Hosmer-Lemeshow analysis.

Model 2: Adjusted for demographics (age, family structure, body mass index, and pensions), the number of comorbidities, the number of medications used, behavioral factors (alcohol and smoking), psychosocial factors (cognitive function, depression, and social support) and physiological factors (ADL and IADL).

## Discussion

In this study, we examined the relationship between a decline in effectance and social participation from the perspective of the number of types of social groups and the type of social participation in a prospective cohort study. Social participation significantly lowered the risk of effectance decline, and this effect increased with an increasing variety of social groups in which subjects participated, regardless of gender. Our results are consistent with prior longitudinal studies of community-dwelling older adults that a higher level of social activity was associated with decreased risk of incident disability in IADL and positive effect did not vary significantly by gender [12], and that participation in a greater number of different organizations reduced the onset of long-term care insurance certification in both males and females [13].

There are 3 plausible relationship mechanisms between social participation and prevention of effectance decline. First, the main effect of social participation is obtained from social relationships. Participation in a broad range of social relationships develops a person’s social network. Social networking ties provide individuals with access to various forms of social support and social networks (e.g., access to material resources, or health-relevant information) [41]. This may enhance subjects’ willingness to take an interest in health-related information through the mass media including newspapers, books, magazines, and television, which promotes the preservation of effectance. Second, stress buffering is also considered a pathway to good health [41]. A prior study [14] has reported that loss of a spouse, which has been classified as one of the most stressful events a person can encounter [42], causes functional decline in the elderly, while social interactions buffer the effects of widowhood on functional decline. Because participation in various social groups can boost social interactions, the elderly who participate in more groups may have more protection against functional decline of stressful experiences, resulting in the maintenance of effectance. Third, the benefits of social participation may be due to the influence of social role. An earlier study has reported that people who maintain a role in social participation experience a lower risk of depression [24]. Participation in social groups may also provide and reinforce meaningful social roles, thereby providing a sense of value and belonging to an older adult’s post-retirement life [43,44]. This sense of attachment to family, friends, and community may provide a strong motivation to maintain functional capacity in later life [16]. As participation in a diversity of social groups increases not only the number of roles, but also the opportunity to acquire meaningful social roles, the elderly who participate in more social groups can sustain stronger motivation to maintain higher-level functional capacity, contributing to lower incident effectance decline.

In relation to the type of social participation, our results have shown that significant associations vary according to gender; among females, participation in all social groups except for senior citizen clubs was inversely associated with a decline in effectance, whereas among males, a beneficial effect on effectance was restricted to hobby groups and volunteer groups. Some prior studies of community-dwelling elderly have investigated gender differences in the effects of social participation on health, and those findings are contradictory. Takagi et al. found that neighborhood-level social participation in 8 types of groups had protective effects on depressive symptoms for women, but not for men [24]. Sun et al. also demonstrated that social integration (i.e., participation in a welfare center association, recreational activities, or a hobby association) enhanced women’s self-rated health, but had no significant effect on men’s health [32]. In contrast, some studies found that social participation produces bigger benefits for the health of males than females [18,19,45]. For example, Lund et al. found a protective effect of social participation (i.e., visiting someone and being visited by someone) on mobility capability for men, but not for women [19]. Our findings are consistent with Takagi et al.’s [24] and Sun et al.’s [32] reports. Because females are more likely to achieve a close relationship from their large and diverse networks than males [46], females may tend to receive positive benefits from social participation, showing that a salutary influence of social participation on effectance is stronger for females than for males.

Of all types of social participation, only participation in senior citizen clubs showed no association with incident effectance decline in both genders. This result is consistent with a previous study that found participation in neighborhood associations or hobby groups to be associated with having more teeth, but there was no association between participation in senior citizen clubs and dental health [28]. The reason for this may be the negative side of social relationships, such as conflict, sense of obligation to the community or burden, and feelings of loss and loneliness [47]. A longitudinal study [48] has suggested that negative social interactions can cause mild cognitive impairment, which is a key risk factor of effectance decline [4]. Regarding the characteristic of social groups which we assessed, senior citizen clubs are targeted for ≥60 aged residents, whereas groups for other than senior citizen clubs have no age limitation [49]. That is, the members of senior citizen clubs are more likely to experience the death of the people they have met through these clubs than through groups without age restriction. Thus, people who participate in senior citizen clubs may have a greater opportunity to experience the feeling of loss characterized by the negative aspects of social networks, resulting in no association between participation in senior citizen clubs and effectance decline. Our findings indicate the possibility that participation in the types of social groups which have an opportunity to come into contact with younger people may help prevent a decline in effectance among community-dwelling elderly.

Our study has several limitations. First, our results are biased by the exclusion of subjects who could not obtain the required valid data or did not return to the questionnaire. Proportions of individuals aged 75 years and older and individuals with poorer ADL were greater in individuals with invalid data than in those with valid data [see Table 1]. Although we do not have any information about the non-responders at baseline, they might have suffered from poor functional capacity or poor social participation, which could have hindered their ability to participate in this study. We speculate that the high-risk population for disability was excluded in this study. It is unclear whether the association between social participation and effectance was overestimated or underestimated. Second, social participation and effectance were assessed by self-report. Therefore, associations among the study variables may be overestimated due to a common response style while several studies have shown that these influences are not as much as one could expect [50]. Third, we did not inquire about the frequency of participation in each social group. The frequency of meetings may vary greatly according to the type of social group [28,49], but the effects of variation in frequency are not reflected in this study. Finally, we cannot rule out the possibility of residual confounding as there could have been several unmeasured parameters such as societal or lifestyle factors that could have influenced the association between social participation and the incidence of decline in higher-level functional capacity. For example, we lacked information on physical activity. Because a prior study suggests that socially active older adults tend to be more physically active and physical activity is associated with a reduced risk of functional disability [51], additional adjustment for physical activity might have changed the observed associations.

In conclusion, social participation may play a role in maintaining effectance among community-dwelling elderly, and this protective effect may be strengthened by participation in a variety of social groups, regardless of gender. Regarding the type of social participation, significant associations vary according to gender, and a salutary influence of social participation on effectance may be stronger for females than for males. These findings suggest that support for community-dwelling elderly that encourages participation in a variety of social groups, taking into consideration their gender in terms of the type of social groups, may be effective for maintaining effectance ability and extending an active, healthy life.
