# Correction: Social Participation and the Prevention of Decline in Effectance among Community-Dwelling Elderly: A Population-Based Cohort Study

**DOI:** 10.1371/journal.pone.0164925

**Published:** 2016-10-18

**Authors:** Kimiko Tomioka, Norio Kurumatani, Hiroshi Hosoi

The authors wish to acknowledge that in the Introduction, Methods and Discussion sections, the authors have re-used some text from their own related PLOS ONE paper and other published sources, either verbatim or with slight modification without the use of quotation marks, and in some cases without citation. Such re-use of the authors’ text and others’ text is not appropriate. The authors wish to deeply apologize for the use of text verbatim from their publication and previous publications and for the lack of appropriate citations and quotation marks.

The list of sources for the re-use of text is as indicated below:

Tomioka K, Okamoto N, Kurumatani N, Hosoi H. Association of psychosocial conditions, oral health, and dietary variety with intellectual activity in older community-dwelling Japanese adults. PLoS One. 2015;10:e0137656. (uncited) [2]

Kanamori S, Kai Y, Aida J, Kondo K, Kawachi I, Hirai H, et al. Social participation and the prevention of functional disability in older Japanese: the JAGES cohort study. PLoS One. 2014;9:e99638. (cited as reference 13 in the published article) [3]

Imai E, Tsubota-Utsugi M, Kikuya M, Satoh M, Inoue R, Hosaka M, et al. Animal protein intake is associated with higher-level functional capacity in elderly adults: the Ohasama study. J Am Geriatr Soc. 2014;62:426–434. (uncited) [4]

James BD, Boyle PA, Buchman AS, Bennett DA. Relation of late-life social activity with incident disability among community-dwelling older adults. J Gerontol A Biol Sci Med Sci. 2011;66:467–473. (cited as reference 12 in the published article) [5]

Miura K, Matsui M, Takashima S, Tanaka K. Neuropsychological characteristics and their association with higher-level functional capacity in Parkinson's disease. Dement Geriatr Cogn Disord Extra 2015;5:271–284. (uncited) [6]

The authors also wish to correct some errors in reference information given in the published article.

Reference 2 of the original published article is incorrect. The correct name of the publisher is: Behavioral Publications. The correct reference is: Lawton MP. Assessing the competence of older people. In: Kent DP, Kastenbaum R, Sherwood S editors. Research planning and action for the elderly: the power and potential of social science. New York: Behavioral Publications; 1972. pp. 122–143. [7]

Reference 50 of the original published article is incorrect. The correct reference is: Lance CE, Dawson B, Birkelbach D, Hoffman BJ. Method effects, measurement error, and substantive conclusions. Organizational Research Methods. 2010;13:435–455. [8]

A closely related study was published in *PLOS ONE* by this author group within the same month (“Association of Psychosocial Conditions, Oral Health, and Dietary Variety with Intellectual Activity in Older Community-Dwelling Japanese Adults” [2]). The reasons for reporting the two related studies in separate papers and re-using portions of text from the other related *PLOS ONE* paper are as follows:

The first paper’s aim was to investigate the comprehensive relationship between psychosocial conditions, oral health, dietary intake, and intellectual activity [2]. Regarding psychosocial conditions, participation in social activities, hobbies, and life worth living were assessed. In the first paper [2], the authors integrated participation in five types of social activities and used them as an indicator of social participation. After the reviewer’s comments about the first paper, the authors thought that it was important to evaluate the association of each type of social participation with intellectual activity. Therefore, the authors examined the relationship between the decline in effectance (corresponding to intellectual activity) and social participation from the perspective of the type of social activities a new study, reported in this paper [1]. Additionally, the first paper is a cross-sectional study, while this paper is a longitudinal study. Therefore, because the two papers have different study aims and designs, it was thought that submitting two separate papers would be appropriate.

Regarding intellectual activity, it is often defined as a form of elderly people’s leisure activities and in fact, there are studies that have categorized the elderly’s leisure activities into cognitive/intellectual activities, social activities and physical activities. However, in the authors’ studies, instead of categorizing intellectual activity as forms of leisure activities, the authors needed an explanation about it being the second highest effectance in the 7 steps of hierarchy model over the elderly’s living function in the Introduction. Both papers required this explanation. The authors tried to avoid re-use and overlap between these 2 papers regarding the explanation of intellectual activity considered as effectance and the reason why the authors gave attention to it as an outcome. However, some text has been re-used in this paper without noting that it was quoted from the first paper [2].

To avoid confusion for the reader, the first paper should have been listed as a reference in this paper. However, because it took a long time for the first paper to be published, the two papers ended up being published closely together. As a result, this paper did not include the first paper as a reference.

The authors also wish to discuss in further detail the closely related study by Kanamori et al. [3] (cited as reference 13 in the original published article).

The similarities between the authors’ study and the Kanamori et al. study are as follows:

1. Regarding social participation, there are cases in which researchers put multiple activities together in one index and those which evaluated each activity individually. The authors chose the latter. Regarding the conditions of participation, one method is to ask for frequency and the other is to ask whether the person has participated or not. The authors chose the latter. The method of evaluating social participation in the authors’ study is the same as the study of Kanamori et al. This is because the authors asked subjects only whether they participated or not and the authors desired to evaluate each activity separately.

The relationship of “number of social participation” and outcome adopted in the study of Kanamori et al. has also been used in many previous studies (Glei DA, Landau DA, Goldman N, Chuang YL, Rodríguez G, Weinstein M. Participating in social activities helps preserve cognitive function: an analysis of a longitudinal, population-based study of the elderly. Int J Epidemiol. 2005;34:864–71.[9], Roh HW, Hong CH, Lee Y, Oh BH, Lee KS, Chang KJ, et al. Participation in physical, social, and religious activity and risk of depression in the elderly: a community-based three-year longitudinal study in Korea. PLoS ONE 2015;10: e0132838.[10], Takeuchi K, Aida J, Kondo K, Osaka K. Social participation and dental health status among older Japanese adults: a population-based cross-sectional study. PloS One 2013;8:e61741.[11], and Zunzunegui MV, Alvarado BE, Del Ser T, Otero A. Social networks, social integration, and social engagement determine cognitive decline in community-dwelling Spanish older adults. J Gerontol B Psychol Sci Soc Sci. 2003;58: S93–S100.[12]). Additionally, there are several studies that have examined the relationship between the number and types of social participation and outcome variables (Kishimoto Y, Suzuki E, Iwase T, Doi H, Takao S. Group involvement and self-rated health among the Japanese elderly: an examination of bonding and bridging social capital. BMC Public Health 2013;13:1189.[13], and Park HK, Chun SY, Choi Y, Lee SY, Kim SJ, Park EC. Effects of social activity on health-related quality of life according to age and gender: an observational study. Health Qual Life Outcomes. 2015;13:140.[14]). Therefore, the authors consider it a general method of researching how social participation affects elderly people’s health and not an original idea coming from Kanamori et al.[3].

2. There are some similarities in the results of the authors’ study and those of the study by Kanamori et al.[3]. The authors quoted the study of Kanamori et al. in the Discussion section, stating that the results of their study were consistent with the findings of Kanamori et al.

The differences between the authors’ study and that of Kanamori et al.[3] are as follows:

1. The outcome of the study conducted by Kanamori et al. is the onset of certification of long-term care insurance, which means basic activities of daily living (BADL). On the other hand, the outcome of the authors’ study is effectance, which is about higher-level functional capacity. The purpose of making effectance the outcome is as follows:

A. A lot of previous studies looking into the impact on functional disability caused by social participation set BADL for their outcome. There are very few previous studies setting instrumental activities of daily living (IADL), which is higher-level than BADL, for their outcome. In fact, the authors do not recognize any study that set effectance, which is second from the highest in the hierarchical model of elderly people’s life function advocated by Lawton, for the outcome. In other words, this is the first article that has examined the relationship between “social participation” and “effectance”.

B. It is known that keeping the ability of effectance prevents deterioration of IADL and cognitive function. Therefore, it is important to identify modifiable protective factors against effectance.

2. Regarding the evaluation of “social participation,” the authors’ study included their own activities (i.e. local event groups). Additionally, the study of Kanamori et al.[3] combined neighborhood associations, in which the frequency of elderly people’s participation is high, and senior citizen clubs to be local community. Kanamori et al. stated in their article that combining neighborhood associations and senior citizen clubs as “local community” was a research limitation. According to other previous studies, many participate in neighborhood associations from an obligatory feeling and it is pointed out that there are few good influences on health. Therefore, in the authors’ study, they evaluated neighborhood associations and senior citizen clubs separately. As a result, among neighborhood associations, only female participants were shown to have gained a preventive effect against lower effectance, while in senior citizen clubs, neither male nor female participants showed any relationship with effectance. In Japan, community-dwelling older adults are recommended to participate in senior citizen clubs to prevent deteriorating BADL. According to the authors’ study, there is no relationship between the participation to senior citizen clubs and sustention of effectance. The point suggested that the possibility of participation in senior citizen clubs does not lead to maintaining intellectual activities is the point advanced on the previous knowledge in the authors’ study. In the Discussion section of the authors’ study, they pointed out that the difference between senior citizen clubs and other social groups is that the former is limited to elderly people and made a proposal that it is possible to prevent deterioration of effectance for community-dwelling older adults by participating in social groups where they can have exchange with younger generations. Furthermore, regarding volunteer groups, Kanamori et al. [3] stated there was no relationship in BADL for either males or females. However, the authors’ study recognized significant relationships for both males and females, and that can be evidence to recommend the community-dwelling elderly, both male and female, to participate in volunteer activities.

3. The authors’ study gives attention to gender difference. The reason for this is that the studies looking into the difference of effect on health by social participation between men and women are not consistent. The studies of both Kanamori et al.[3] and James et al. [5] regarding the effect of preventing deterioration of BADL and IADL through social participation stated that there were no differences in the positive effect between genders. The authors quoted the results of Kanamori et al.[3] and James et al. [5] in the beginning of their Discussion section.

The authors’ study classified social participation by the contents of each activity and investigated the relationship of participation in each group and effectance. The authors could show results that the effect of preventing deterioration of effectance is greater for females than males. According to the previous studies, there was no difference between males and females regarding the preventive effect of BADL/IADL deterioration through social participation. Therefore, the authors’ study showing the possibility that the effect of preventing deterioration of effectance is greater on females than males is different from previous studies, including that of Kanamori et al., and significant.

4. It is meaningful that the authors’ study had a higher response rate than the study of Kanamori et al. (72.5% vs. 46%).

## References

[1] Tomioka K, Kurumatani N, Hosoi H (2015) Social Participation and the Prevention of Decline in Effectance among Community-Dwelling Elderly: A Population-Based Cohort Study. PLoS ONE 10(9): e0139065 10.1371/journal.pone.0139065 26406326 PMC4583439

[2] Tomioka K, Okamoto N, Kurumatani N, Hosoi H. Association of psychosocial conditions, oral health, and dietary variety with intellectual activity in older community-dwelling Japanese adults. PLoS One. 2015;10:e0137656 10.1371/journal.pone.0137656 26360380 PMC4567331

[3] Kanamori S, Kai Y, Aida J, Kondo K, Kawachi I, Hirai H, et al Social participation and the prevention of functional disability in older Japanese: the JAGES cohort study. PLoS One. 2014;9:e99638 10.1371/journal.pone.0099638 24923270 PMC4055714

[4] Imai E, Tsubota-Utsugi M, Kikuya M, Satoh M, Inoue R, Hosaka M, et al Animal protein intake is associated with higher-level functional capacity in elderly adults: the Ohasama study. J Am Geriatr Soc. 2014;62:426–434. 10.1111/jgs.12690 24576149

[5] James BD, Boyle PA, Buchman AS, Bennett DA. Relation of late-life social activity with incident disability among community-dwelling older adults. J Gerontol A Biol Sci Med Sci. 2011;66:467–473 10.1093/gerona/glq231 21300745 PMC3055280

[6] Miura K, Matsui M, Takashima S, Tanaka K. Neuropsychological characteristics and their association with higher-level functional capacity in Parkinson's disease. Dement Geriatr Cogn Disord Extra 2015;5:271–284. 10.1159/000381333 PMC4521071 26273243

[7] Lawton MP. Assessing the competence of older people In: Kent DP, Kastenbaum R, Sherwood S editors. Research planning and action for the elderly: the power and potential of social science. New York: Behavioral Publications; 1972 pp. 122–143.

[8] Lance CE, Dawson B, Birkelbach D, Hoffman BJ. Method effects, measurement error, and substantive conclusions. Organizational Research Methods. 2010;13:435–455.

[9] Glei DA, Landau DA, Goldman N, Chuang YL, Rodríguez G, Weinstein M. Participating in social activities helps preserve cognitive function: an analysis of a longitudinal, population-based study of the elderly. Int J Epidemiol. 2005;34:864–71. 10.1093/ije/dyi049 15764689

[10] Roh HW, Hong CH, Lee Y, Oh BH, Lee KS, Chang KJ, et al Participation in physical, social, and religious activity and risk of depression in the elderly: a community-based three-year longitudinal study in Korea. PLoS ONE 2015;10: e0132838 10.1371/journal.pone.0132838 26172441 PMC4501692

[11] Takeuchi K, Aida J, Kondo K, Osaka K. Social participation and dental health status among older Japanese adults: a population-based cross-sectional study. PloS One 2013;8:e61741 10.1371/journal.pone.0061741 23613921 PMC3629217

[12] Zunzunegui MV, Alvarado BE, Del Ser T, Otero A. Social networks, social integration, and social engagement determine cognitive decline in community-dwelling Spanish older adults. J Gerontol B Psychol Sci Soc Sci. 2003;58: S93–S100. 12646598 10.1093/geronb/58.2.s93 PMC3833829

[13] Kishimoto Y, Suzuki E, Iwase T, Doi H, Takao S. Group involvement and self-rated health among the Japanese elderly: an examination of bonding and bridging social capital. BMC Public Health 2013;13:1189 10.1186/1471-2458-13-1189 24341568 PMC3878629

[14] Park HK, Chun SY, Choi Y, Lee SY, Kim SJ, Park EC. Effects of social activity on health-related quality of life according to age and gender: an observational study. Health Qual Life Outcomes. 2015;13:140 10.1186/s12955-015-0331-4 26361977 PMC4566195

